# Global Political Logics and Mainstream Discourses on Illness in the Declarations of the State of Exception in the Context of the Covid-19 Pandemic: The Case of the USA, France, and Spain

**DOI:** 10.1007/s10912-024-09856-y

**Published:** 2024-06-26

**Authors:** Mar Rosàs Tosas

**Affiliations:** PSITIC Research Group at Blanquerna School of Health Sciences, Ramon Llull University, Barcelona, Spain

**Keywords:** Agamben, Autoimmunity, Covid-19, Democracy, Derrida, Esposito, Illness narrative, State of exception, Triumph narrative

## Abstract

At the outbreak of the Covid-19 pandemic, several countries declared “states of exception,” that is, authorized legal devices that, in the face of circumstances deemed catastrophic, permit the implementation of extraordinary measures and the temporary suspension of some rights in order to restore the previous state of affairs as soon as possible. This paper offers a comparative textual analysis of the different states of exception declared in the USA, France, and Spain. I argue that these texts constitute *a privileged site to explore how prevalent global political logics and mainstream discourses on illness are interwoven*. Regarding the global political logics in play, I hold that these declarations constitute an instantiation of democracy’s autoimmune character; it attacks itself in order to protect itself. Regarding mainstream discourses on illness, I explore how illness is regarded as a threat to one’s *self* (by something seemingly *other*) and the notion that therapy must consist of securing the self’s *triumph* over anything seemingly other. This twofold analysis reveals that an aporetic dialectic between self and other—as regards politics and illness—operates in these declarations, most likely because it is, in fact, one and the same dialectic, upon which Western epistemology rests. Furthermore, I suggest that these texts *reflect* and *promote* these dominant logics, contributing to shape human relationships around the globe in a certain dangerous way.

## Introduction

The goal of the present paper is to examine comparatively the texts of the declarations of the state of exception that followed the outbreak of the Covid-19 pandemic in the USA, France, and Spain—this being a first approach to these texts, and with the wish to, soon, broaden the sample by including the declarations of other countries and, thereby, offering a more global view on the phenomenon.

The reason why I pick this object of study is that, in my view, these declarations of the state of exception constitute *a privileged site to explore both prevalent global political logics and mainstream discourses on illness, and the way they are interwoven*.

When it comes to the global political logic, which I will address in sections 1 and 2, I will try to show that the declarations of the state of exception constitute an example, an instantiation of a phenomenon extensively studied by some political philosophers, that democracy is immune to itself, that is, autoimmune; it attacks itself in order to protect itself (Derrida 2003; Derrida 2005; Esposito 2013).

Interestingly, the term “autoimmune” is also proper to illness, to biomedicine (Esposito 2013; 59). In section 3, I will focus, then, on the role that common discourses on illness in the West play in the texts of the declarations of the states of exception—in particular, how illness is regarded as a threat to one’s self (by something seemingly other) and therapy must consist of securing the self’s triumph over anything seemingly other.

In short, I want to explore how in the declarations of the state of alarm there is an aporetic dialectic between self and other at stake, both when it comes to politics and illness—most likely, because it is one and the same dialectic, upon which Western epistemology rests.

The importance of such an exploration stems from the fact that these texts not only *reflect* these dominant logics, but also *promote* them. In other words, these texts *render visible* in a synthetic, condensed way, the aforementioned logics, and, as we shall see, serve as platforms that *promote* them and *spread* them even more by contributing to shape human relationships around the globe in a certain way. That is, the logics and discourses that operate in these declarations are not exceptional ideas to be found exclusively in these texts, but extremely common ideas to which citizens in the USA, France, and Spain—and beyond—have been constantly exposed à propos Covid-19 and its severity. In a way, this is a feature of the Covid-19 pandemic that distinguishes it from the Great Flu: despite its severity, the severity of the 1918 pandemic was downplayed by official institutions and public actors (Barry 2004, 335; Jurecic 2012, 4; Kolata 1999, 51-54). By way of contrast, all citizens around the globe have been exposed to discourses concerning the serious, far-reaching implications of the Covid-19 pandemic.

The reasons to have chosen USA, France, and Spain as case studies are the following. As explained above, my goal is to point out that a very similar logic affected the texts of the declarations of state of exception. I want to study this similarity *despite the contexts in which these texts were written being different in several aspects*; i.e., the legal frames regulating the states of exceptions of these three countries are very different, as I will explain below, and the degree to which citizens have free access to healthcare is also considerably different. At the same time, it was important to pick three countries that share a relevant feature: that had been transformed by the cultural movement known as Enlightenment, which promoted the notion of the individual that I want to explore in the texts of the declarations of the state of exception. This implied, then, picking three Western countries in which the Enlightenment had left a lasting footprint—it is the case of France, the USA, and Spain.

## Section 1. Is the state of exception so exceptional?

In *Political Theology I*, originally published in 1922, jurist Carl Schmitt—German, Nazi—studied the phenomenon of the state of exception. As he argued, in their constitutions, most countries, including democracies, envisage the possibility of the declaration of a state of exception—a legal device that, in the face of an exceptional, catastrophic threat, allows for a temporary suspension of some rights in order to restore normality as soon as possible, which when adhered to the regular legal frame would not allow for the re-establishment of normality.

In a way, a year before, Walter Benjamin (1999) had also written about the value of the state of exception. For Benjamin, the state of exception was a transitory state that must be overcome so that something new, and better, opens up, while for Schmitt (1985), the value of the state of exception stems from its capacity to safeguard the order in force, to preserve power. In other words, for Schmitt, the state of exception belongs to the juridical order, outside of which there is only chaos. For Benjamin, by contrast, the state of exception is a mechanism that helps to overcome the juridical order, inside of which there is only oppression and guilt, and not freedom and justice.

The discussion between Benjamin and Schmitt opened up a debate about the value of the state of exception, a debate in which several contemporary philosophers have taken part. Some of them, writing in the first half of the twentieth century, are Jewish intellectuals that, in an original fashion, combine libertarian utopias in vogue at that time with the Jewish messianic tradition, in which the category of the state of exception plays a crucial role. But the debate on the value of the state of exception for political theory also includes intellectuals that, writing since the 1990s onwards, make a more heterodox use of theology and the category of state of exception to inform their critique of certain political logics, such as Taubes (1993), Badiou (2003), Derrida (1989–1990), and Agamben (2005). Among them, Agamben stands out for the number of books and shorter texts he devoted to this figure. In *Homo Sacer I* (1995) and *State of Exception* (2005), Agamben develops the idea that the state of exception is a threshold of indistinction between the inside and the outside of the law (Agamben 1998)—neither legal nor illegal. He describes this threshold as a force-of-law—crossing out *law*—a state in which the law keeps its validity; that is, it is “in force”, but does not signify anything in particular; that is, it does not translate into concrete, identifiable laws. It is mere potential to use Aristotelian jargon. It constitutes the originary moment, the zero moment, of the law. This “validity without significance” is the *Geltung ohne Bedeutung*—in German—with which Gershom Scholem, the scholar of religions, in a letter to his friend Walter Benjamin in the 1930s, referred to the status of the law in Kafka’s work *The Trial*: the law is in force, but does not translate into identifiable laws. As a result, the law can neither be grasped nor avoided, and is therefore suffocating. In Scholem’s words, this is:

[a] state in which revelation appears to be without meaning, in which it still asserts itself, in which it has validity but no significance [in dem sie gilt, aber nicht bedeutet]. A state in which the wealth of meaning is lost and what is in the process of appearing (for revelation is such a process) still does not disappear, even though it is reduced to the zero point of its own content, so to speak. (1989, 142)

For Agamben, this threshold of indiscernibility between the inside and the outside of the law is not an accidental, or exceptional, phenomenon that takes place only in catastrophic circumstances, but a logic that lies at the very core of Western sovereignty. It is a terrible logic, Agamben insists: in order to protect citizens, they are deprived of some rights. That is, in order to be protected, they are rendered vulnerable. And to him, each declaration of a state of exception makes this logic clear, manifest: concrete laws are suspended, but not the force of law.

Agamben criticizes the proliferation of states of exception following 9/11, designed as devices for the “war on terror.” Turning to Benjamin’s 8th thesis of the philosophy of history, he goes so far as to claim that the exception has become the rule and that this is leading the West toward a global civil war (Agamben 2005, 52-57). And he proposes to overcome this logic of a permanent state of exception by switching off the force of law—but it is unclear to me how to perform this operation.

The Covid-19 pandemic is the second event—after the US-led global war on terror—that has triggered massive declarations of states of exception all over the world. I am going to focus on the declarations of exception declared in Spain, France, and the USA.

## Section 2. Protecting while rendering vulnerable

Before addressing these declarations, it is worth pointing out when they were declared in the USA, France, and Spain, and under which legal umbrella. Article 116 of the Spanish Constitution regulates the three existing types of state of exception—state of alarm, state of exception, and state of war. Among the three, the state of alarm is the one declared in less severe circumstances. It might be declared due to natural catastrophes, for example, owing to serious pandemics or shortage of first need products. It was the one declared by the Spanish president on March 14, 2020. It might be declared for a maximum of 15 days, after which it might be prolonged, as was the case. Within Spanish contemporary democracy, it had only been declared once—in December 2010, when it was declared à propos an illegal strike of air traffic controllers.

In France, the “exceptional measures” were initially taken within the legal frame of the Code of Public Health (Article L3131-1). But on March 23rd, a “state of sanitary emergency” was created for cases “of *sanitary* catastrophe endangering, by its nature and severity, the population’s health.” This new regulation, created ad hoc, is specific for threats to the health of citizens, which clearly distinguishes this new regulation from the general French “state of emergency” dating from 1955. The state of emergency created in 1955 allows for the suspension of some freedoms, such as freedom of circulation, and for the delivery of some weapons. It was created à propos the war in Algeria and declared then. It has been declared twice more ever since—the last time is because of the risk of terrorist attacks in 2017.

In the case of USA, there are multiple laws at stake. Its Constitution grants certain “emergency powers” in the article on executive power; i.e., the Congress may authorize certain measures. But mayors and state governors can also declare states of emergency in their territory—because of heavy rains, for example. I will only refer to the two national emergency declarations issued by US President Donald Trump on March 13th—one under the Stafford Act, usually associated with natural disasters, and another under the National Emergency Act.

In all three countries, the declarations coincide in the beliefs that the situation is “exceptional,” that the problem is a “severe threat,” that measures need to be taken “urgently,” and that they will be “temporary.” To counteract its negative effects, these declarations promote the implementation of certain measures—which, needless to say, have a history (Ruiz-Domènec 2020; Vigarello 1993). The nature of most measures allowed by these declarations is also fairly similar—i.e., limitation of mobility, limitation of mass gatherings and travel, non-essential business shutdowns, turning to on-line education when possible, quarantine for those who might be infected with Covid-19, lockdowns, and mask mandates. The differences between the measures allowed by these declarations lay, rather, on the moment, the place, and the intensity of its application, and on some aspects that have to do with the idiosyncrasy of each healthcare system—i.e., one of the measures in the USA had to do with waving requirements to get free access to healthcare, which does not make sense in Spain, where free access to the public healthcare system is guaranteed to all individuals.

Although providing an exhaustive list of all the measures described in or allowed by these declarations falls beyond the scope of the present paper, it is worth pointing out that they all aspire to three different and complementary goals/aims. First, they aspire at bringing to a close, or at least slowing, the spread of the virus (US). The Spanish text reads “to contain the virus” and “to hold back the pandemic” (Real Decreto 2020, 3, 12). The French text, in turn, presented its goal as “to eradicate the sanitary catastrophe [...], bring it to a close” (Code de la santé publique 2020, 2). And the declarations from the USA spoke about “containing and combating the virus” (Proclamation 2020, 1) and “stemming” the pandemic (Letter from President Donald. J. Trump 2020, 1).

Second, these declarations aim at guaranteeing that citizens will be provided with the best healthcare assistance possible—ultimately, then, to maximize the health of the population. In this sense, the French text insists on the need to “prevent and limit the potential threats to the health of the population” and to “guarantee public health” (Code de la santé publique 2020, 1). The Spanish text emphasizes the urgency to “mitigate the sanitary [...] impact of the virus” (Real Decreto 2020, 3) and the US one urges to prevent Covid-19 from “straining the healthcare system” (Proclamation 2020, 1)—and this is one reason, among others, that explains the need to “waive requirements” to access the healthcare system.

The third complementary goal of these declarations is to provide relief—beyond strictly sanitary measures—to those affected by the virus. The Spanish text puts it as follows: it wants to “mitigate the [...] social and economic impact of the virus” (Real Decreto 2020, 1,12). The US text, in turn, proposes to waive tax deadlines, for example.

What concerns us is: how do these declarations present the ultimate operation they seek to perform?

It could be put this way: to protect citizens and the current order of things as much as possible from the impact of the pandemic, to “bring to a close” (France) this “threatening” disorder (Spain), and to resume the previous order of things as fast as possible. It is a matter of national and economic “security,” in terms of the US texts, and it is the only way to protect fundamental rights, which are “at risk,” according to the Spanish view.

In short, these texts are an instantiation of two facts, already described by Italian philosopher Giorgio Agamben (2005). First, the function of the state of exception is not to open up something different, but to safeguard the order in force. And second, the state of exception in order to protect the order in force, and the “regular” lives of individuals within this order, deprives them of some rights, rendering them vulnerable. This logic does not only affect the state of exception, but democracy as a whole: one of the foundations of democracy, the so-called social contract, shares the same logic—I agree to lose some freedoms in order to be protected from others. Derrida put it in the following words: democracy is autoimmune, in order to protect itself, it undermines itself. Or, put differently, it can only protect itself by undermining itself.

The ultimate foundation of this logic is to be found in Hobbes—in order to be protected from others, we need to grant part of our freedom to the sovereign. At this point, Roberto Esposito’s analysis of current politics worldwide proves enlightening. For him, communities, in order to protect themselves, design an immune system consisting of a number of protective devices in the form of norms. But, unfortunately, he argues, this defensive system, this immune system has intensified so much that it no longer protects the community, but undermines it (Esposito 2013, 127). The logic at stake is no longer immunological, but autoimmunological—it destroys the very body it sought to protect. In his own words: “[l]aw constitutes community through its destitution. It does so, by extreme paradox, exactly insofar as it seeks to strengthen its identity, to ensure its mastery over its own identity” (Esposito 2011, 22). For Esposito, this logic is indebted to Hobbes and Locke insofar as it rests upon the belief that the individual has to *protect* one’s interests from others, and therefore has to protect oneself, rather than *expose* oneself to others and *take care* of them (Esposito 2013, 128). A certain notion of the self underlies this view, then: the Hobbesian individual is sovereign to himself, while the one that Esposito (and so many others) defends is conceived as intersubjective—as structurally open to the influence of others and as inclined towards others. In short, Esposito criticizes Hobbesian individualism as a type of metaphysics that hinges on an individual “enclosed in his own absoluteness” (Esposito 2013, 17).

The declarations of the state of exception insist on the mutual influence of citizens as well as the need to care for citizens—a version of Esposito’s intersubjective self seems at stake here. But at the same time, the texts insist on the need to protect citizens from an excess of this influence—here, a rather Hobbesian self seems at stake.

Explored through this lens, the declarations of the state of exception reveal a last contradiction, or aporia, of democracy: in order to take care of ourselves and of others we need to separate ourselves from others, because their influence on us and our influence on them might be dangerous. That is, we need to guarantee what we now call “social distancing,” not only on a physical level, but also on an epistemological one. Obviously, this logic is made more visible when the threat at work is a virus. But the same logic lies at the heart of democracy. This point would deserve a further exploration that cannot be developed in the context of the present essay. But to put it in few words, Western democracies rest upon the Universal Declaration of Human Rights, which enumerates and describes a number of *individual* rights that need to be protected from potential threats among which the intromission of others occupies a prominent place. In other words, the texts of the declarations of the state of alarm use a notion of the individual as sovereign of itself, with clear boundaries, and insist on the need to protect it from others, just as democracy does in general. The porosity of the individuals makes them vulnerable, and hence the need to patrol their boundaries with legal devices such as the state of exception.

Let’s leave for a moment the domain of politics and move now to the domain of illness. In fact, it is telling that Esposito draws attention to the fact that the term “immune” is proper both to the legal field, to be immune is to be exempt from the application of some rules, as happens in a state of exception, and the biomedical field, to be immune to a disease is not to be potentially affected by, or exempt from the threat of, a disease (Esposito 2013, 59).

## Section 3. Triumphing over illness

In this section, I attempt to demonstrate that the so-called triumph illness narratives, that circulated broadly during the pandemic, are haunted by a logic of autoimmunity, just as it happens with the state of exception and democracy in general, as seen in section 2.

In the last decades, anthropology and sociology of health, together with narratology, have studied illness narratives—the way patients, healthcare practitioners, family, friends, and administration *explain* disease, and how these narratives influence the way they *experience* disease. And among their findings, two in particular shed light on our analysis of the declarations of the state of exception.

First is the fact that Western citizens tend to prefer listening to what Arthur Frank (2013) labeled “restitution narratives”—that is, narratives that focus on the possibility of recovery and that, therefore, focus on positive signs, emphasize “positive thinking” and silence and avoid the expression of negative feelings. Within these types of narratives, the subject is not to be transformed by illness, but is supposed to defend itself against the disease, to fight to avoid being defeated (changed) by it, and ultimately to be successful, which is to win this battle. This is why Ann Jurecic (2012) refers to restitution narratives as “triumph narratives.”

Elsewhere, I studied how, within this type of illness narrative, illness itself is presented as a “state of exception” that needs to be left behind (Rosàs Tosàs 2019). This supposes that here was a previous, normal order, and it needs to be recovered. In my view, the texts of the declaration of state of alarm we have examined participate in exactly the same logic. And in several of its fragments, they do so by turning to military vocabulary, which, as Susan Sontag famously examined, is also a characteristic of illness narratives—the fight against cancer. In the US texts, we need to “successfully [...] combat the virus” (Proclamation 2020, 1). In the Spanish one, we need to “fight an emergency” (Real Decreto 2020, 4). And during the speech in which he declared the state of exception, the Spanish president described healthcare practitioners as a “shield against the virus”—as if to say, with all this ammunition, everything is going to be alright. Thus, the triumph narrative regarding the national response to the pandemic is articulated with military rhetoric.

Moreover, just as the pandemic is presented in these declarations as a phenomenon that *disrupts normality*, in triumph narratives, sick people are commonly presented as a challenging *threat to normality*. This threat to normality is to be understood not only in biomedical terms, but also in socioeconomic terms: the sick, as long as they enact the “sick role”—the famous expression coined by sociologist Talcott Parsons (1951)—cannot perform “normally: they are exempt from some duties, such as working, and deserve some special rights, such as being taken care of and, in some contexts, having a right to disability pay.” In short, the sick disrupt normality, and this is why they tend to be perceived as a burden. This is also the same logic that informs the declarations of the state of exception we have examined: they aspire to patrol the boundaries of normality.

The second finding from anthropology of health I wanted to refer to is that, in the West, most illness narratives—whether created by patients, physicians, family, or administration—insist on the distinction between the “self” that is sick and the illness. This might seem pretty obvious in the case of illnesses that are provoked by pathogens. But, according to anthropologists, this distinction is made across the entire spectrum of illness. According to most illness narratives, a person *is not* his or her cancer, but *has* cancer, as something external to his or her self. Illness is presented, then, as something that, coming from without the self, disrupts the self. In fact, the ultimate reason why we are supposed to be able to “triumph over illness”—the first idea I just pointed out—has to do with the fact that illness is perceived as separate from us.

According to this typically Western discourse, then, to preserve health is to preserve one’s self against the intromission of otherness. The discourses regarding infectious diseases with severe consequences are the example par excellence of this logic: we need to preserve our physical self from them. But this discourse has a contradiction, as historian of health Georges Vigarello points out à propos plagues: plagues accentuate the need to preserve this border precisely because they make clear that this border is not so clear-cut. In his words, “[t]he black death intensified the image of the porosity of organic borders, presenting the body as more permeable” (1993, 56). This porosity was threatening. And because of this, the awareness of this organic blurriness between self and other led to an apparently contradictory conclusion: the insistence on the need for a strict boundary between self and other. In Vigarello’s words: “The black death suddenly contributed to better imagining the slow elaboration of a dam between the body and its milieu” so that “purity” was preserved (1993, 56).

However, this distinction between one’s self and the illness is not that easy to hold, although it is constantly deployed. For example, autoimmune diseases collapse this distinction—one attacks oneself. Or some writers—sometimes in poetry—express the surprise of *being* their illness (Camps Mundó 2010). And, therefore, when they (their self) are being hugged, their illness is also being embraced.

This problematic dichotomy between self–other and interior–exterior articulates the texts of these declarations. What informs them is the *fear of contagion* by the other—the Hobbessian protection from the other, as pointed out before. What they seek is to preserve *purity*—the notion that anthropologists have defined as a lack of contact with otherness. At this point, it is worth recalling Foucault words: “The plague is the moment when the spatial partitioning and subdivision (quadrillage) of a population is taken to its extreme point, where dangerous communications, disorderly communities, and forbidden contacts can no longer appear” (Foucault 2003, 47).

Given this dichotomy between self and other, it is natural to argue that we have to fight together *against* the virus, as the Spanish text reads. At this stage, though, it is clear that *the virus is not the sole other*. In these discourses, there are two types of others at stake—the virus and the people who might have it—but at times, they are presented as one and the same other. And their aggressive interplay is reinforced by the dichotomy us–them—a “pathogen,” “nationwide pandemic” that “has been introduced into our country from abroad,” reads the US text, and hence the measures to “suspend entry of foreign nationals,” preventing them from coming and “touching” us (Letter from President Donald. J. Trump 2020, 1).

## Conclusive remarks

This dichotomy is problematic in two senses. First is because of the global dimension of the virus and the global dimension of socioeconomic interactions. One sentence by the Spanish president while explaining he was going to declare a state of alarm highlights this tension between “us,” “them,” and the fact that the phenomenon is global: “the battle against the virus that all countries fight”—he said, instead of “we all fight.” And second is because this notion of the self as clearly separated from the other—so common in illness narratives in general, and also fundamental in the texts of these declarations—fails to acknowledge one of the most common assumptions of contemporary philosophy of the self that the distinction self–other is always in deconstruction and that the self is fundamentally traversed by and constituted by others, by the environment, and by the milieu. Consequently, if we insist so much on separating one’s self from others—as the texts of these declarations push us to do—one’s self will vanish for epistemological reasons, for psychoanalytic reasons, for mental health reasons, and for socioeconomic reasons.

In other words, the only way to secure the existence of both the self and the others seems to imply acknowledgement that they are not two entities with distinct boundaries. This is why Esposito celebrates the bursting of a self-sufficient self. That is, instead of trying to preserve the self against immunitary and autoimmunitary attacks, Esposito celebrates the fact that the sovereign self is being dismantled by them. Michael Lewis (2015), a lucid reader of Esposito, explains why: so much immunological apparatus to protect from the other leads to an autoimmunitary process, Esposito claims, as explained above. But Lewis warns the reader of the fact that, for Esposito, this autoimmunity might be understood “either as a militaristic defense against the foreign, or as an hospitable relation to the other” (2015, 222). In the first case, autoimmunity is deemed negative as long as it seeks to preserve the self’s self-sufficiency. By contrast, the second way of understanding autoimmunity might lead to a fertile outcome, because it constitutes “the origin of a breach in the supposedly impermeable boundaries of the individual which opens that individual self to its ‘other,’ rendering the immune individual inherently *communal*, which is to say *political* in its very organismic life” (Lewis 2015, 214). Ultimately, this is why, for Esposito, the autoimmunitary logic at work in global politics does not have to be *toppled*, but *traversed*: the current autoimmunitary processes might be a path for the self to become aware of the fact that the relationship between self and other is so intricate that, ultimately, one cannot be distinguished from the other. And, therefore, self and other could treat each other with so much hospitality that, in the end, neither of them knows who is at whose home.

The declarations of the state of exception examined reveal a notion of the self that is diametrically opposed to Esposito’s. In my view, the problematic dialectic that these declarations render visible—there is only self if it is kept at a distance from the other, but that distance undermines the self—is the dialectic that Esposito criticizes in the Hobbessian model. And it is, as well, the fundamental contradiction that is constitutive of democracy and that might be insurmountable. I say “of democracy”—that is, of democracy as a whole, not only in the USA, France, and Spain—consciously. Although the declarations of the state of exception we have explored need to be declared by each *national* government, a distinctive feature of the Covid-19 pandemic is the *global* impact of the decisions taken concerning the virus, argues historian Ruiz-Domènec in his study of the epidemics in history with more sociocultural effects. After all, our analysis reveals that the discourses informing the declarations of the state of exception in the USA, France, and Spain boil down to the same logics. Our guess is that it is a matter of a global logic to be found in the texts that regulated the states of exception declared because of the pandemic. Further research is needed to test this hypothesis. It must be noted, though, that even if further research demonstrated that the same notion of the individual informs the texts of other countries regulating the states of exception, it does not mean that the impact of the pandemic was the same in all of them. It is clear that the *measures* taken by governments, the *moment* they were implemented, and their *impact*, varied greatly from one country to another (Cheng, Barceló, Hartnett et al. 2020).

As might be clear at this stage, I embrace Esposito’s proposal of a self that is structurally open to the other, in the theoretical plane, but I have to admit that, in practice, I cannot fathom how the texts of the declarations of the state of emergency could have been informed by Esposito’s notion of the self.
